# Supplementary material for: Learning curve and surgical outcome of robotic assisted colorectal surgery with ERAS program
Source: Sci Rep. 2022 Nov 29;12:20566. doi: 10.1038/s41598-022-24665-w (PMC9709162; doi:10.1038/s41598-022-24665-w)
Supplement: Supplementary file 2 — Supplementary Information 2. [file 41598_2022_24665_MOESM2_ESM.zip › RAL Raw data-Ñ[▒K/115/OP N_ñw╝╨░O▒Kñσ.pdf]

W52 / W52

臺中榮民總醫院
手術室護理記錄

[redacted]

基本資料

- 室別： 開刀房-17室，手術科別： CRS，手術類別： 常規，日期： 2021/10/07
- 進入時間： 0725 ； 麻醉開始時間： 0745 ； Time Out時間： 0920 ； 劃刀時間： 0921 ； 手術結束時間： 1349 送出時間： 1410 ；
- 麻醉方式： GE

  診斷：Malignant tumor of rectum

  手術名稱：Robot Assisted Low Anterior Resection

  手術局部用藥：nil

  術前預防性抗生素：
  - 抗生素名稱一： Veterin 1GM IVD/由麻醉護理人員給予
  - 時間： 0905
  - 上次簽章: 林家羽

  ，追加預防性抗生素：
  - 抗生素名稱一： Veterin 1GM IVD/由麻醉護理人員給予
  - 時間： 1305
  - 上次簽章: 黃彥翔
- 手術醫師： 蔣鋒帆，陳瑨夫，陳凱祥
- 麻醉醫師： VS 陳永銘
-
  - 刷手護士A ： 黃彥翔，時間： 0725，接替刷手一： 劉雅汶，時間： 1150
  - 流動護士A ： 林家羽/歐真勤，時間： 0725，接替流動一： 黃彥翔/歐真勤，時間： 1150

皮膚狀況

A-V Shunt： 無

術前： 完整

記錄者： 上次簽章: 林家羽

術後： 不完整

- 1.紅腫

部位(術後)：

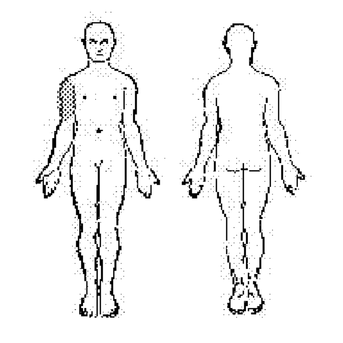

右上臂0.5*5cm壓紅可反白

記錄者： 黃彥翔 上次簽章: 黃彥翔

手術準備

手術臥式 單一手術臥式

-
  - 手術臥式： 仰臥
  - 使用附件： 溫毯 38 ℃，頭圈，墊肩，其它： 腳架*2
  - 皮膚準備： 10%Aq Betadine，Hibiscrub，2% Chlorhexidine
  - 電燒： 使用電燒: 電極片位置： 其它： 電燒墊，雙極電燒，記錄者： 上次簽章: 林家羽

止血帶T： 無

植入物

- 植入物名稱/號碼/數量/部位： Covidien GIA 80mm-3.8mm*1
  Davinci Xi EndoWrist Stapler 45mm-4.3mm*2/Retum

, 記錄者： 黃彥翔

標本送檢

- 細菌培養 無
- 細胞學 無
- 冰凍切片 無
- 病理標本
  組織名稱一： rectum
  ,
  組織名稱二： IMA ROOT
  ,
  組織名稱三： distol cut end
- 截肢 無

標本交付 標本： 3 袋 , 標本： 0 盒 , 標本： 0 瓶 , 細胞學： 0 管 , Culture Tube： 0 管 , 被交付醫師： CR 陳璿夫 ,

- 記錄者： 上次簽章: 黃彥翔
- 時間： 1327

傷口術後敷料

傷口敷料：

- 請於圖上標示部位：

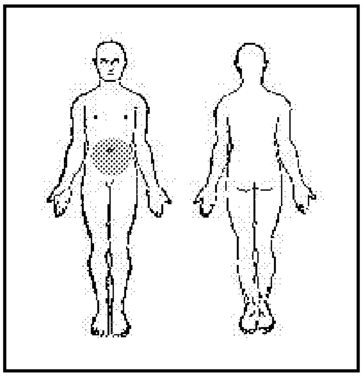

- 傷口敷料： mepore

導管

導管	天數	導管資訊	插入日期	插入時間	更換日期	拔除日期	拔除時間	拔除原因	預計拔除/更換	預計拔除/更換日期
Double Lumen CVC(1)	1	號碼:8Fr ; 部位:; 象限:RU; 註記:15cm;	2021/10/07	0820						
Foley(1)	1	號碼:16 Fr.; 部位:膀胱; 註記:10cc;	2021/10/07	0815						

用物計數

- 手術中出血量： minimal c.c.
- 手術中尿量： 700 c.c.
-
  - 其它： 無

用物計數： 多切口同時縫合

- 1.器械： 正確 , 2.縫針： 正確 , 3.刀片： 正確 , 4.紗布： 正確 , 5.腹部墊： 未使用 , 6.紗球： 未使用 , 7.PEANUTS ： 未使用 , 8.紗條： 未使用 , 9.絲帶： 未使用 , 10.針頭： 未使用 , 11.Vessel loop： 未使用 , 12.Boots： 未使用 , 13.Jelco： 未使用 , 14.棉條： 未使用 ,
  - 第一次：
    - 刷手護士 ： 劉雅汶
    - 流動護士： 上次簽章: 黃彥翔

        - 時間： 1240

,

  - 第二次：
    - 刷手護士 ： 劉雅汶
    - 流動護士： 上次簽章: 黃彥翔
    - 時間： 1245

,

  - 第三次：
    - 刷手護士 ： 劉雅汶
    - 流動護士： 上次簽章: 黃彥翔
    - 時間： 1330

備註

備註： Davinic scopy30 度 No.1

- 記錄者： 上次簽章: 林家羽
- 時間： 0725

填寫護理人員 黃彥翔

正式摘要

W52 / W52

doctorRank:B
